# The NIST Detector-Based Luminous Intensity Scale

**DOI:** 10.6028/jres.101.014

**Published:** 1996

**Authors:** C. L. Cromer, G. Eppeldauer, J. E. Hardis, T. C. Larason, Y. Ohno, A. C. Parr

**Affiliations:** National Institute of Standards and Technology, Gaithersburg, MD 20899-0001

**Keywords:** calibration, candela, illuminance, lumen, luminous intensity, lux, measurement, photometer, photometry, scale, standards, units

## Abstract

The Système International des Unités (SI) base unit for photometry, the candela, has been realized by using absolute detectors rather than absolute sources. This change in method permits luminous intensity calibrations of standard lamps to be carried out with a relative expanded uncertainty (coverage factor *k* = 2, and thus a 2 standard deviation estimate) of 0.46 %, almost a factor-of-two improvement. A group of eight reference photometers has been constructed with silicon photodiodes, matched with filters to mimic the spectral luminous efficiency function for photopic vision. The wide dynamic range of the photometers aid in their calibration. The components of the photometers were carefully measured and selected to reduce the sources of error and to provide baseline data for aging studies. Periodic remeasurement of the photometers indicate that a yearly recalibration is required. The design, characterization, calibration, evaluation, and application of the photometers are discussed.

## 1. Introduction

Traditionally, standardization in photometry was a discipline driven by primary light sources, first candles, then flames [1], carbon-filament lamps, and, beginning in 1948, blackbody radiators operated at the freezing-point temperature of molten platinum [2]. The latter marked a turning point, because the platinum-point blackbody, valued for its reproducibility and universality compared with the earlier alternatives, was the first standard photometric source with radiometric properties that could be readily calculated, in principle.

Over time, dissatisfaction with platinum-point blackbody standards grew. For the few national laboratories that had them, they were difficult to maintain. They operated at a temperature of little technological interest [taken first as 2045 K, later 2042 K, on the International Practical Temperature Scale of 1968 (IPTS-68)], and the applicability of this broadband radiation to spectroradiometry was poor. In 1975, Blevin and Steiner [3], reflecting the mood of the period, made two proposals. They sought first to redefine the photometric base unit in a manner to fix its relationship with other Système International des Unités (SI) base units, such as the meter and the ampere. Second, they argued that the photometric base unit should be changed from the candela to the lumen, considering the close relationship between luminous flux (lumen, lm)^2^ and radiometric power measurements (watt, W).

After additional study and due consideration, in 1979 the 16th Conférence Générale des Poids et Mesures (CGPM) adopted the first of these proposals. They abrogated the definition of the candela (originally called the new candle) first adopted by the 8th Conférence Générale in 1948, and redefined it as follows [4]:

The candela is the luminous intensity, in a given direction, of a source that emits monochromatic radiation of frequency 540 × 10^12^ hertz and that has a radiant intensity in that direction of (1/683) watt per steradian.

The 1979 redefinition of the candela permitted diverse methods to be used in deriving luminous intensity scales. All the methods also rely on the principles governing photometry as compiled by the Bureau International des Poides et Mesures (BIPM) for the Comité Consultatif de Photométrie et Radiométrie (CCPR) [5]. These include the Commission Internationale de L’Éclairage (CIE) spectral luminous efficiency function for photopic (cone) vision, *V*(*λ*), which relates visual sensitivities at different wavelengths [6]. (The lone frequency of 540 × 10^12^ Hz mentioned in the definition has a wavelength of 555.016 nm in standard air, which for almost all purposes can be taken to be 555 nm without affecting the accuracy of a real measurement.)

Since the redefinition, national standards laboratories [7–14] and other research facilities [15] have been free to realize the candela by use of whatever radiometric means they found most suitable. Most have used detectors that were equipped with filters that were designed to match their spectral responsivity to the *V*(*λ*) function. At NIST [then the National Bureau of Standards (NBS)] the luminous intensity scale remained based on a standard source, a blackbody radiator operating at the freezing-point temperature of molten gold (the gold point) [7].

**Fig. 1:**
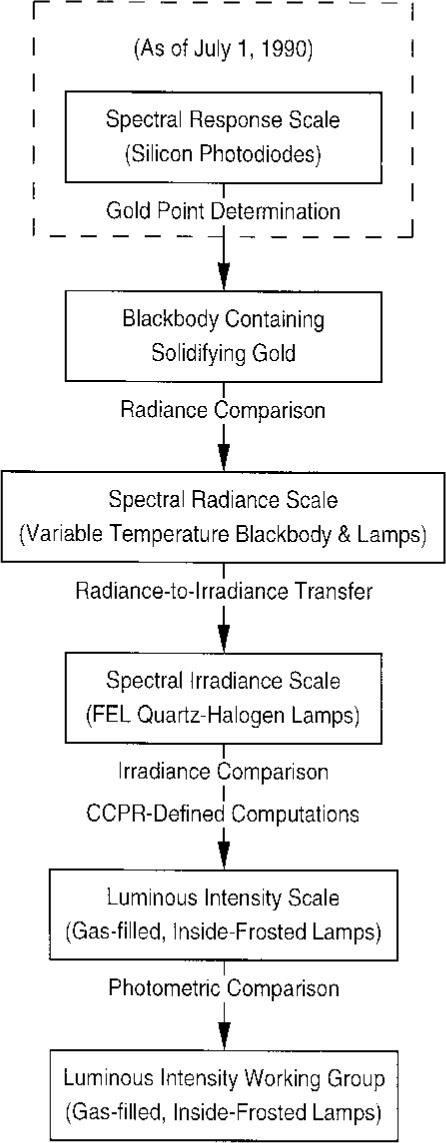
Calibration chain for luminous intensity prior to the present study.

As shown in Fig. 1, the blackbody radiation at the gold point (1337.58 K on IPTS-68) was used to calibrate a variable temperature blackbody, which provided the NBS scale of spectral radiance [16]. From this the spectral irradiance scale was derived [17]. The luminous intensity scale was derived through spectral irradiance measurements of selected lamps forming a primary reference group, which maintained the candela with respect to the spectral irradiance scale. A secondary reference group of lamps, calibrated against the primary group, was used for routine candela calibrations.

All the measurements in this lineage compared a light source with another light source. The final measurement uncertainty of 0.8 % (2 standard deviation estimate) [18] contained a relatively large component from the uncertainty in the gold-point temperature at the top of the chain (comparing IPTS-68 with thermodynamic temperature), and it was further limited by the long-term behavior of the incandescent lamps that were used.

In 1990, the introduction of the new International Temperature Scale (ITS-90) caused changes. The gold point was redefined as 1337.33 K [19], which caused the NIST luminous intensity scale to shift, depending on the color temperature of the source, by approximately 0.35 % [20]. More important, NIST revised its procedures to decouple the spectral radiance scale from ITS-90. NIST now considers the gold-point temperature to be a measured rather than a defined quantity. While the current NIST measurement of 1337.33 K ± 0.23 K (restated from “3*σ*” to *k* = 2) [21] is in exact agreement with ITS-90, the new policy allows for the possibility of future scale revisions as experimental information becomes available. The current NIST gold-point temperature of 1337.33 K is detector based. That is, the result follows from measurements using absolute radiometric detectors, a silicon photodiode and an electrically calibrated radiometer.

The purpose of this paper is to describe the considerable simplification that results by realizing the candela against the detector base directly. We expand upon our previous reports on this subject [22,23], giving more details behind the new NIST scale for luminous intensity and discussing our experience with it. The benefits of this conversion include reduced uncertainty in our calibration services and the additional flexibility to provide new calibration services for detector-based devices.

## 2. Experimental Approach

### 2.1 Mathematical Framework

The photometric analog of power in radiometry is luminous flux, *Φ*_v_, (lm), where

$$\Phi_{v}=K_{m}\int_{\lambda }\Phi_{e}\left(\lambda \right)V\left(\lambda \right)d\lambda ,$$ (1)

where *Φ*_e_(*λ*) is the spectral radiant flux of the light (W/nm) and *K*_m_ is the proportionality constant in the definition of the candela. While a strict reading of the definition gives *K*_m_ = 683.002 lm/W [6], for almost all purposes it is taken to be 683 lm/W without affecting the accuracy of any real measurement.

A photometer is a device that can be used to help measure such a flux. Typically, it has an output current^3^ *I* (ampere, A), where

$$I=\int_{\lambda }\Phi_{e}\left(\lambda \right)s\left(\lambda \right)d\lambda ,$$ (2)

where *s*(*λ*) (A/W) is its spectral responsivity. It is advantageous to factor

$$s\left(\lambda \right)=s\left(555\right)s_{n}\left(\lambda \right),$$ (3)

where *s*(555) (A/W) is the value of *s*(*λ*) at 555 nm. This emphasizes the similarity of *s*_n_(*λ*) to *V*(*λ*), both dimensionless functions that are normalized at 555 nm. It also permits the overall uncertainty of the spectral responsivity scale to be associated with one number, *s*(555), with the function *s*_n_(*λ*) consisting of relative measurements only.

The luminous responsivity [24] of the photometer is *s*_v_ (A/lm), where

$$s_{v}=\frac{I}{\Phi_{v}}=\frac{s\left(555\right)}{K_{m}}\frac{\int_{\lambda }\Phi_{e}\left(\lambda \right)s_{n}\left(\lambda \right)d\lambda }{\int_{\lambda }\Phi_{e}\left(\lambda \right)V\left(\lambda \right)d\lambda }.$$ (4)

For a perfect photometer, *s*_n_(*λ*) would equal *V*(*λ*), and its luminous responsivity would be independent of the power distribution of the light. In practice, this approach requires knowing *Φ*e(*λ*) in order to calculate a spectral mismatch correction factor

$$F=\frac{\int_{\lambda }\Phi_{e}\left(\lambda \right)V\left(\lambda \right)d\lambda }{\int_{\lambda }\Phi_{e}\left(\lambda \right)s_{n}\left(\lambda \right)d\lambda }.$$ (5)

In general, the closer *s*_n_(*λ*) is to *V*(*λ*), the better *F* will be known for the same incertitude about *Φ*_e_(*λ*).

**Fig. 2:**
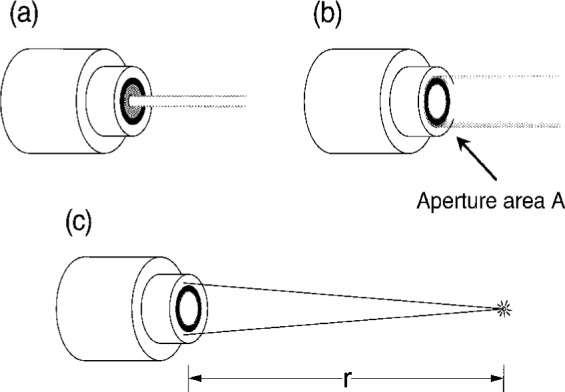
Application of a photometer to luminous intensity measurement as a progression. (a) When the light beam underfills the entrance aperture, the photometer measures luminous flux (lm), the photometric analog to radiant power. The responsivities of our detectors were tested in at least seven positions, as shown. (b) When the light beam overfills the entrance aperture, the photometer measures illuminance (lx). (c) When the photometer is used with a point light source at a distance, the aperture area and the distance to the source combine to define a solid angle. The photometer then measures the luminous intensity (cd) of the source.

Figure 2 illustrates the application of such a photometer to luminous intensity measurement. In Fig. 2a, it is supposed that the photometer intercepts a beam of light, and that all the light illuminates only a portion of the active area of the photometer. In this case, the photometer would have an output current *I* from which the luminous flux of the beam could be determined, presuming that *s*(*λ*) is sufficiently invariant from point to point over the active area:

$$\Phi_{v}=\frac{K_{m}FI}{s\left(555\right)}.$$ (6)

In Fig. 2b, it is further supposed that the photometer is fitted with an aperture of precisely known area. Then, if the light is not confined to a small spot but rather overfills the aperture uniformly, the photometer would have an output current *I* that is proportional to the illuminance *E*_v_ (lumen per square meter, lux, lx) on the aperture. For an aperture area *A* (square meter, m^2^),

$$E_{v}=\frac{K_{m}FI}{s\left(555\right)A}.$$ (7)

Figure 2c shows the overall geometry for luminous intensity measurement. A point light-source a distance *r* from the plane of the aperture and lying on the normal to its center would have a luminous intensity *I*_v_ (lumen per steradian, candela, cd), where

$$I_{v}=\frac{K_{m}FIr^{2}}{s\left(555\right)A}.$$ (8)

The applicability of these geometric prerequisites to real measurements is explored below.

### 2.2 Description of the Photometers

To measure photometric quantities and to maintain the luminous intensity scale at NIST, a group of eight photometers has been developed. Many laboratories have used absolute detectors such as electrically calibrated thermal detectors and self-calibrated silicon photodiodes to realize the candela. We chose to use calibrated silicon photodiodes because of their wider dynamic range and simplicity of operation. The photometers contain specially selected silicon photodiodes with *V*(*λ*) matching filters, as well as the electronics to implement the high-sensitivity, wide-dynamic-range circuit previously described [25]. With an integration time of 1.67 s, a measurement bandwidth of 0.3 Hz, and an amplifier gain of 10^11^ V/A, the output voltage noise in these devices corresponds to ≈ 1 fA of photocurrent. This important feature of the NIST detectors permits precise measurement of *s*_n_(*λ*) even in the regions where its values are small.

**Fig. 3:**
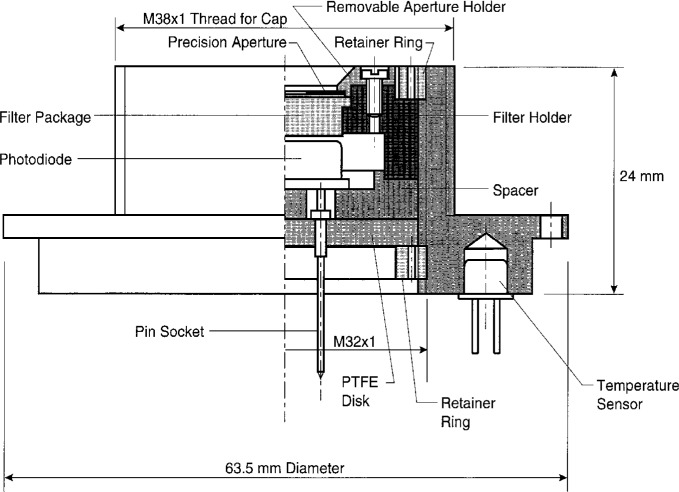
Photometer design. A filter modifies the spectral responsivity of a silicon photodiode to replicate as closely as possible the 1924 CIE spectral luminous efficiency function for phototopic vision.

Figure 3 depicts the photometer design. The silicon photodiode, the *V*(*λ*) correcting-filter package, and a precision aperture are mounted in the front piece of a cylindrical housing. A PTFE^4^ disk of low electrical conductivity supports the photodiode; small pin-terminals in the disk form a socket. The *V*(*λ*) filter is glued to a holder and is positioned close to the photodiode. On the front side of the filter, the precision aperture is glued to a holder. This holder is carefully machined so that its front surface, the frontmost surface of the photometer, is 3.00 mm from the plane of the aperture knife edge. All these components are marked in a manner that permits us to preserve their orientation during disassembly and reassembly.

The cylindrical housing itself, which extends back from the front piece shown in Fig. 3, contains an amplifier that also acts as a photocurrent-to-voltage converter. A switch selects the transimpedance gain of the amplifier, decade values from 10^4^ Ω through 10^10^ Ω. (Photometers 1 and 2 also have 10^11^ Ω ranges.) The characteristics of the filter and photodiode change with temperature, so the operating temperature of the photometer is monitored by a sensor inserted in the front wall of the housing [26]. The housing contains all additional components necessary for signal and temperature outputs; it is lighttight and acts as an electrical shield.

### 2.3 The New Luminous Intensity Scale

**Fig. 4:**
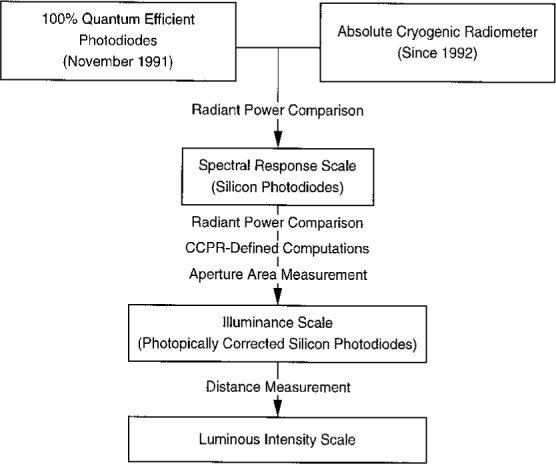
Calibration chain for luminous intensity as revised by the present study.

It is simpler to realize the candela by this approach, diagramed in Fig. 4. The luminous intensity scale is derived by measuring *s*(*λ*) of each photometer in the group directly against the NIST spectral responsivity scale. The spectral responsivity scale is derived from comparative measurements against absolute radiometric detectors; at the time of the initial study, 100 % quantum efficient detectors [27] were the basis of the scale. Today, the scale is based on cryogenic radiometry^5^ [28]. With the application of the *V*(*λ*) curve in Eq. (5), and the application of the geometric definitions in Eq. (8), the candela is determined. Additionally, since the photometers do not age in use as rapidly as lamps do, an additional step to form a working group of photometers for routine use is unnecessary.

## 3. Characterization of the Photometers

### 3.1 Instrumentation and General Procedures

**Fig. 5:**
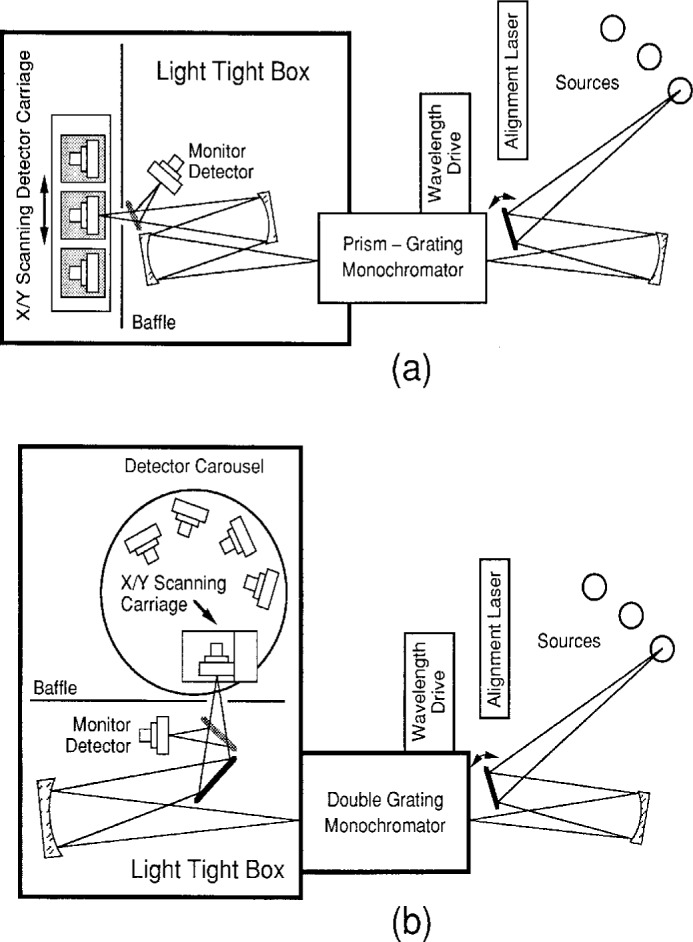
Facility used to calibrate the photometric detectors: (a) with visible and IR radiation, (b) with UV radiation.

The principal apparatuses used to study the photometers and their components are shown in Fig. 5. They comprise the Spectral Comparator Facility (SCF), which holds the NIST spectral responsivity scale referenced in Figs. 1 and 4. An ultraviolet (UV) instrument spans 200 nm to 400 nm; a visible/near-infrared (IR) instrument spans 350 nm to 1800 nm. A detector under test is held in a carriage that can be translated under computer control. Any point on the active area of the detector can be positioned at the focus of a nearly circular spot, 1.1 mm or 1.5 mm in diameter for the visible or UV system, respectively. The carriage also holds reference detectors that serve as secondary standards and that are measured alternately with the device being tested. Compensation for changes in the light source during the course of the measurement is made by using the signal from a monitor detector. The computer controls the monochromator, which has a bandpass of 4 nm for this spot size and a spectral standard uncertainty of ± 0.2 nm [29]. The apparatuses typically deliver a few microwatts of optical power to the detector.

Before the photometers were assembled, the SCF was used to study their components, both to diagnose systematic effects and as the basis for aging studies. When the spectral responsivity of an individual photodiode or a photometer (the photodiode, filter, and aperture together) was measured, the device itself was mounted on the carriage. For the spectral transmittance of a filter alone to be determined, the filter was held on the carriage, but a photodiode behind it was not. (Filter transmittance is the ratio of the apparent detector responsivity with and without the filter interposed in the beam.) In this case, the photodiode was tilted to prevent interreflections.

Care was taken to insulate thermally the devices from the carriage, which heats up during use because of its stepping motors. The ambient temperature during measurement was monitored; when applicable, the temperature circuitry of the device under test was used. This permitted a direct comparison between the temperatures at calibration and use. Generally, variations in ambient temperature were held within ± 1 °C during the course of a measurement.

In addition to the optical calibrations performed at the SCF, the transimpedance gains of the photometer amplifiers were calibrated electrically. With this procedure, the photodiode is replaced by a computer-controlled voltage source, *V*_IN_, and a resistor substitution box in series. Unlike the internal resistors *R*_f_ built into the photometer heads, the external resistors *R*_EXT_ are easily remeasured. (As explained in Ref. [25], *R*_f_ is the transimpedance gain of the amplifier.) For many combinations of internal and external resistors (as selected by the photometer gain switch and the substitution box, respectively), the output of the photometer, *V*_OUT_, is measured for a series of *V*_IN_. The linear coefficient of this dependence, as obtained from a least-squares fit, is equal to the corresponding *R*_f_/*R*_EXT_. This permits the individual values of *R*_f_ to be determined with a relative expanded uncertainty of < 0.01 % by data fitting. Calibrations on the SCF, reported in the unit volt per watt for an individual photometer gain-switch setting, can be transferred between different settings when these data are used.

### 3.2 Photodiodes

For this project we used Hamamatsu S1226 and S1227 series photodiodes^6^ [30]. They were selected for the largest shunt resistance that the manufacturer could provide, 2.5 GΩ to 7.0 GΩ, in order to minimize noise and drift in the circuit [25]. This type of photodiode has less infrared sensitivity than some others, which is advantageous for photometry. As a consequence, their infrared response is more temperature dependent than the alternatives. We used quartz rather than glass or resin windows, since we found that the former had less surface scatter. S1227-1010BQ photodiodes having 1 cm^2^ area were used in Photometers 1 and 2 because they contained larger *V*(*λ*) filters. The other six photometers used S1226-8BQ 0.3 cm^2^ photodiodes, with the exception of Photometer 4, which contained an S1227-66BQ. (The only difference was in the case.)

**Fig. 6:**
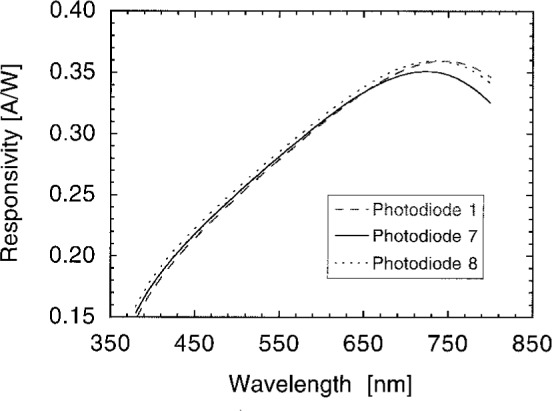
Absolute responsivity of the silicon photodiodes used in the detectors. The dashed curve is Photodiode 1, type S1227-1010BQ. Photodiode 2, of the same type, matches very closely. The solid curve is Photodiode 7 and the dotted curve is Photodiode 8, both of type S1226-8BQ. All other photodiode curves are bounded by the latter two and are similarly shaped. The relative standard uncertainty of 0.3 % is commensurate with the curve widths.

Figure 6 shows the absolute spectral responsivity of three of these photodiodes, at one spot in their centers, as measured at the SCF. The dashed curve is the measurement of Photodiode 1. Photodiode 2 behaved similarly. The solid and dotted curves, measurements of Photodiodes 7 and 8 respectively, bound the responsivity curves of the remaining photodiodes.

**Fig. 7:**
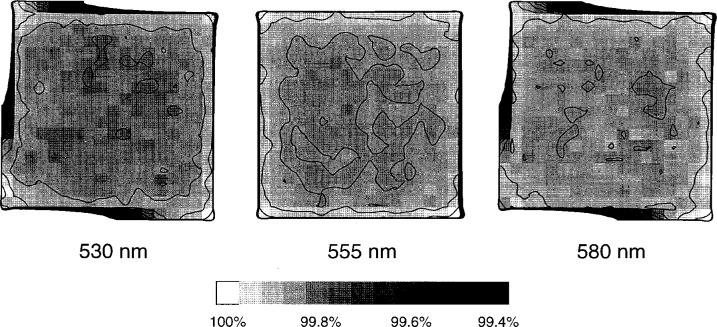
Responsivity map of a typical photodiode used in this study. The responsivity of a photodiode (A/W) was measured while scanning a monochromatic probe beam over the surface. This photodiode, which was used in Photometer 2, is 1 cm on a side. The grey scale shows the responsivity at a point, referenced to the greatest value on the device (100 %). The contours indicate changes of 0.05 % in responsivity.

The eight photodiodes were chosen after screening many more for uniformity over their active areas, particularly the portion that would be visible through an aperture. Uniformity maps such as the one shown in Fig. 7 for Photodiode 2 were made for each device. To construct a uniformity map, the photodiode responsivity was measured on the SCF on a grid of points 0.5 mm apart at three different wavelengths. Mathematica [31] was used to generate surface plots. Typically, the greatest responsivity was at the edge of the photodiode, as in Fig. 7 where the most sensitive spot is the lower right corner. The responsivities over the interior “bowl” of the selected photodiodes were generally constant to better than 0.2 %.

**Fig. 8:**
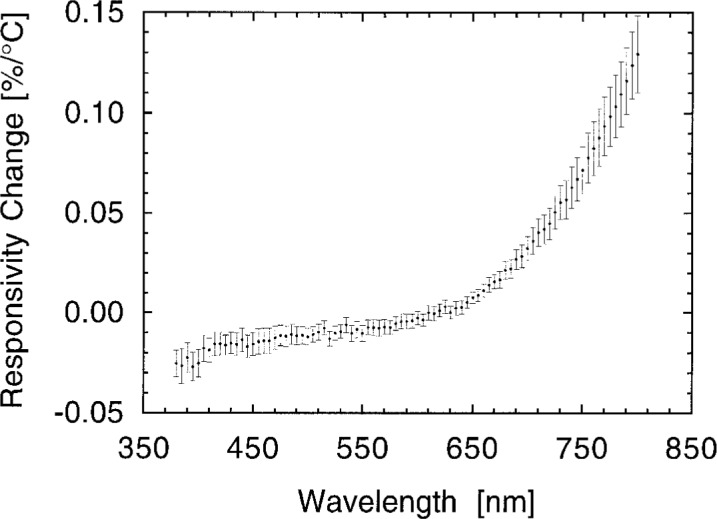
Temperature dependence of the silicon photodiodes at 30 °C. Responsivities of six photodiodes of the types used in this work were measured at 25 °C, 30 °C, and 35 °C. The plot shows the linear change in responsivity, as a fraction of their nominal values, averaged over the six photodiodes. Individual variations among the six generally agreed within the measurement noise. The error bars represent the statistically estimated standard deviation, from the sample of six.

The change in photodiode responsivity due to a change in temperature is shown in Fig. 8. Six photodiodes, most of which were included among the final eight, were tested in a temperature-controlled housing. At each wavelength, the spectral responsivities of the six were measured at the SCF at 25 °C, 30 °C, and 35 °C. Figure 8 shows the average of the six results, the linear temperature dependence as determined through least-squares fitting. For the wavelengths of most interest in photometry, 400 nm to 700 nm, the temperature dependence of the photodiode responsivity was < 0.03 %/°C.

### 3.3 Filters

We obtained layered, colored glass filters from various sources to benefit from the experience that this diversity offers. Filters 1 and 2 were provided through the courtesy of the National Research Council of Canada (NRC), Filter 3 was provided courtesy of the National Physical Laboratory of the U.K. (NPL), and Filters 4 to 8 were manufactured by PRC Krochmann (PRC)[32]. Such filters are individually made to achieve a good realization of the *V*(*λ*) function. First, the glasses are carefully chosen [8,33], and then the thicknesses of the individual glass layers are determined through an iterative procedure including repeated polishing and transmittance measurements. Filters 1 and 2 were originally designed to match QED-200 trap detectors; Filter 3 was designed to match Centronics OSD 300-5 photodiodes. Filters 4 to 8 were optimized to match our type of silicon photodiode.

While spectral match is important, so that Eq. (5) is insensitive to *Φ*_e_(*λ*), other important filter properties include the spatial uniformity, birefringence, and temperature dependence. Filters 4 to 8 were selected from among 24 candidates after visual inspection. Filters with obvious dislocations, scratches, bubbles, and other optical defects were rejected. The remaining filters were screened for uniformity by scanning them with a white-light spot 1.5 mm to 2.0 mm in diameter. Those with the sharpest and largest changes were eliminated.

Since the filters are composed of dissimilar layers cemented together, any resulting strains might cause birefringence or a polarization-dependent transmittance. (The light from a monochromator during calibration is partially polarized.) To verify the absence of such a problem, representative filters were tested. A plane polarizer was interposed between the photometers and a lamp operating at approximately 2856 K. No change in signal above noise was noted as the photometer was rotated, limiting the potential error to 0.01 %. Nevertheless, candidate filters that showed the greatest birefringence were also rejected.

After selecting the most promising filters, more detailed diagnostics were performed. Transmittance measurements were made in 5 nm intervals, and at many positions on the filters to determine their spatial uniformity. Hexagonal patterns were used, consisting of 37 spots for the larger filters (1 and 2), and 7 spots for the smaller (3 to 7). Figure 9 shows the average transmittances of all spots measured on representative filters, using the SCF. Figure 9a compares representative filters from the different sources; others from a common source would be indistinguishable on the graph. However, Filter 8 was from a different batch and provided a better spectral match than the other PRC filters. The small difference between it and the others is highlighted in Fig. 9b.

Figure 10 shows the variation among the measurements at the different spots, expressed as the scatter of the measurements. Scatter in excess of the measurement noise (the heavy curves) represents non-uniformity in the filter transmittance. Figure 10b provides a striking illustration of how the individual layers in these filters contribute differently at different wavelengths. Below 525 nm, the change in transmittance between Filters 5 and 8 (seen in Fig. 9b) is well correlated with the improved uniformity of Filter 8.

**Fig. 11:**
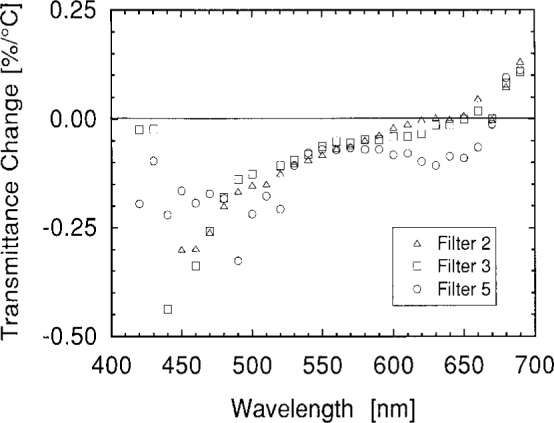
Temperature dependence of the *V*(*λ*) matching filters. Trans-mittances at 23 °C and 33 °C were measured using a Cary 2390 spectrophotometer. The small differences plotted are of the same magnitude as the uncertainties in the measurements—this data is shown to illustrate the overall trend. △, Filter 2 (NRC); □, Filter 3 (NPL); ○, Filter 5 (PRC).

Of particular concern is the temperature dependence of the filter transmittance. Figure 11 shows representative data obtained by using a commercial spectrophotometer equipped with a sample heater. A 3 mm by 10 mm probe beam was used. For Filter 3, this data is consistent with the filters discussed in Ref. [8]. This data is also consistent with the broadband temperature dependence of the complete photometers, which is discussed in detail below.

### 3.4 Apertures

The photometers were fitted with precision apertures, nominally 0.5 cm^2^ for Photometers 1 and 2, and 0.1 cm^2^ for Photometers 3 to 8. They were electroformed out of nickel-clad copper and given a black, nickel finish. The fabrication and properties of similar apertures are discussed in Ref. [34]. Most important to us is the resultant knife-edge from this process, sharp and without burrs. However, such apertures may depart from circularity.

The Precision Engineering Division at NIST measured and certified the areas using a View Engineering Precis 3000 vision-based measuring machine [35]. After a pass was made to find the approximate center of the aperture, 720 radii were measured from the center to the lip at 0.5° angular intervals. The measurements were not sensitive to the method of lighting the aperture (i.e., different forms of front and back lighting). The area was estimated from these radii by a polygonal approximation. The combined standard uncertainties of the radii measurement and the area estimation were given as 0.02 % for the larger apertures and 0.05 % for the smaller. Since the coefficient of linear thermal expansion for copper is ≈ 0.0017 %/°C, temperature corrections were unnecessary.

### 3.5 Assembled Photometers

After the photodiode, filter, and apertures were individually tested, they were assembled into photometers as shown in Fig. 3. The advantage to calibrating the components assembled is that internal reflections and scattering have similar effects during both calibration and use. The essential role of the SCF is to calibrate the spectral responsivity *s*(*λ*) of the photometers to determine *s*(555) [Eq. (3)] and *F* [Eq. (5)]. The small output spot from the SCF can be positioned at various places within the aperture.

The first attempt at calibrating the photometers was to measure *s*(*λ*) at seven positions within the aperture, comprising the vertices and center of a regular hexagon. (Photometers 1 and 2 were measured at 37 positions, which formed a larger, regular hexagonal pattern.) The average over these positions was taken to be *s*(*λ*) for the photometer as a whole. However, consistency among the photometer calibrations was improved by a factor of two by using the following method.

*s*(*λ*) was first measured at 5 nm intervals at one position near the center of the aperture of each photometer. Data from representative photometers are shown in Fig. 12a. Of particular importance in these data is the degree of IR and UV suppression, the latter including both transmission and fluorescence signals.

However, a correction was needed because *s*(*λ*) varied over the aperture area. The spectral responsivity of each photometer, relative to the center point, was determined at 50 nm intervals on a fine, rectangular mesh of points. For the larger apertures (Photometers 1 and 2) the step size was 0.25 mm; for the smaller apertures (Photometers 3 to 8) the step size was 0.2 mm. Measurements that were not affected by the aperture edge were averaged.

Figure 12b shows such data, the ratio of the average responsivity to the responsivity of the center spot. Polynomial fits are made to these data in order to interpolate between them. This permits us to estimate the average responsivity, given the center point responsivity, at all wavelengths. After application to the data in Fig. 12a, the final spectral responsivities for representative photometers are shown in Fig. 12c. The scatter given in the lower part of the figure is only the statistical noise of measuring *s*(*λ*) at the center. Additional uncertainties also apply, and they are discussed below. During the calibration process the temperature of a photometer was monitored using its built-in thermometer. Variations were generally held to ± 1 °C. The average temperature was recorded for each photometer to be used for temperature dependence corrections.

**Fig. 13:**
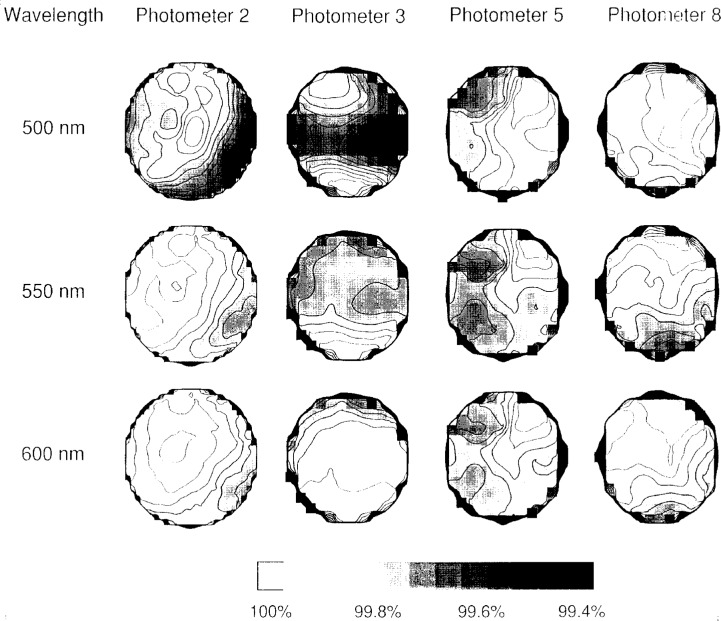
Responsivity map of representative photometers. The responsivities (A/W) of the photometers were measured while scanning a monochromatic probe beam over the aperture area. Photometer 2 had an aperture diameter of 7.98 mm; the others had a diameter of 3.57 mm. The grey scale shows the responsivity at a point, referenced to the greatest value measured (100 %). The contours indicate changes of 0.05 % in responsivity.

Figure 13 shows the mesh of spectral responsivity measurements in more detail. Photometer 3 provides a striking illustration of how spatial nonuniformities may be associated with the individual glass layers in a filter, each affecting a particular wavelength band. This data also helps to estimate the systematic error that might arise if the aperture is not fully and uniformly illuminated during a measurement.

**Fig. 14:**
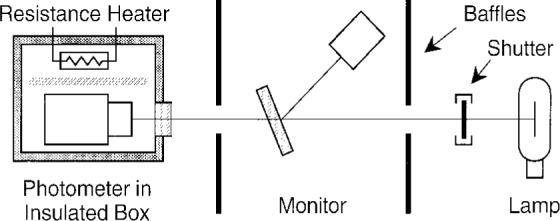
Arrangement to determine the overall temperature dependence of the photometers. The photometer was allowed to reach thermal equilibrium overnight in an insulated box also containing a resistance heater. The photometric responsivity of the photometer was then measured, using a temperature-controlled detector to compensate for variations in the reference lamp.

*s*(*λ*) varies with the temperature of the photodiode and the filter, as shown in Figs. 8 and 11. We measured the overall temperature effect by operating representative photometers at elevated temperatures. Figure 14 diagrams the experimental setup. A photometer was placed in a heated, plastic foam box and left to reach thermal equilibrium overnight. It was illuminated in the normal manner by an inside-frosted lamp of the type formerly used at NIST for luminous intensity calibrations. The lamp had a color temperature ≈ 2856 K. A temperature-controlled monitor detector with a *V*(*λ*) filter was used to compensate for the variation in lamp output from lighting to lighting.

**Fig. 15:**
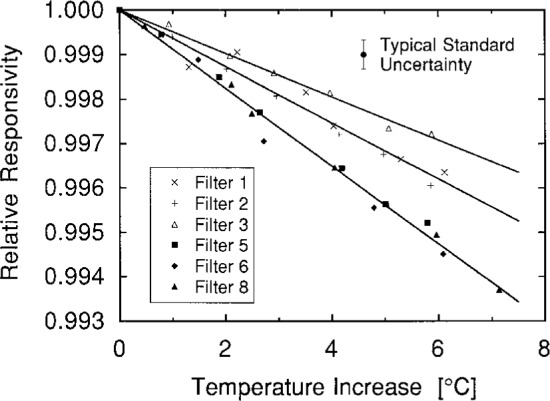
Temperature dependence of photometer luminous responsivity when viewing a broadband source at 2856 K. Photometer number (filter source): △, 3 (NPL); ×, 1 (NRC); +, 2 (NRC); ■, 5 (PRC); ♦, 6 (PRC); ▲, 8 (PRC). Linear fits include all data from each filter source.

Figure 15 shows the results. The luminous responsivity of the photometers decreased with increasing temperature, as measured with each photometer’s built-in thermometer. As expected, the data form clusters that depend on the filter construction. Therefore, all data concerning filters from the same source are considered together and fit to a common line. Compared with the value when the photometer was unheated, the responsivity of Photometer 3 decreased by 0.049 %/°C, the responsivities of Photometers 1 and 2 decreased by 0.063 %/°C, and the rest decreased by 0.088 %/°C. The standard uncertainty of these results is < 0.002 %/°C. The temperature effect would be different when measuring sources with other spectral compositions.

Direct comparison of these results with the data of Fig. 11 is difficult because of the large uncertainties in the latter. Nevertheless, the spectral temperature dependence presented in Figs. 8 and 11 corresponds to broadband changes (as above) of 0.08 %/°C, 0.06 %/°C, and 0.10 %/°C, respectively. The largest discrepancy is for Photometer 3. Ref. [8] gives an independent measurement of 0.12 %/°C for a similar photometer.

Pertinent aspects of the photometers are summarized in Table 1. As explained in Ref. [25], the higher the shunt resistance of the photodiode, the better can be the signal-to-noise ratio of the circuit. A limiting photocurrent noise ≈ 1 fA in Photometers 1 and 2 corresponds to a sensitivity limit ≈ 10^−7^ lx. Besides the spectral correction factor *F*, a traditional metric of the match of *s*_n_(*λ*) to *V*(*λ*) is *f*_1_′ [24], which is also shown in the table.

### 3.6 Illuminance Uncertainty

Following Eq. (7), the relative combined standard uncertainty, *u*_c,r_, of the illuminance responsivity *I*/*E*_v_ of the photometers arises from the standard uncertainties of *s*(555), *F*, and *A*. They are summarized in Table 2. By adopting the terminology of the BIPM [36] and ISO [18], the uncertainties are categorized as Type A, meaning those that were evaluated from the statistics of repeated measurements; and Type B, meaning those that were not (such as estimates of possible systematic effects based on scientific judgment). These uncertainties are reported in relative (that is, fractional) form, as percentages, because of the way the uncertainties scale and combine in Eq. (7).

The principal uncertainty in *s*(555) is that of the NIST spectral responsivity scale. The currently accepted relative standard uncertainty of 0.11 % [37] arises largely from the uncertainty in the absolute spectral responsivity of silicon photodiode trap detectors, with smaller additional contributions resulting from comparisons between the trap detectors and the working standards. The uncertainty that arose from random effects in comparing the photometers with the scale, obtained by averaging the standard uncertainties shown in Fig. 12c for the eight photometers, is 0.04 %.

Calculation of *F* [Eq. (5)] requires knowledge or presumption of the spectral distribution of the source, *Φ*_e_(*λ*). Since the photometers are normally illuminated by an incandescent lamp operating with a color temperature of 2856 K (CIE Source-A), we begin by presuming Planckian distributions. Following Eq. (3), only the uncertainty of *s*_n_(*λ*) relative to the NIST scale matters. The statistical noise of the responsivity measurements is shown in Fig. 12c. After adding their effects in quadrature, the resultant uncertainty of the *Φ*_e_(*λ*)-weighted integral in *F* is 0.01 %. (This result presumes that the possible error that is accounted for under the 0.11 % spectral responsivity scale uncertainty is uniform for all wavelengths. If it varies with wavelength, the possible error in *F* may be greater than 0.01 %. Nevertheless, for the purpose of analysis of the combined uncertainty in illuminance calibration, this effect is accounted for by the spectral responsivity scale uncertainty already in the budget.)

An uncertainty is also introduced from the correction polynomials, which do not pass directly through the data points in Fig. 12b, and which slightly differ in additional ways from the exact correction functions. This is, at worst, a 0.01 % effect. Further, there is an uncertainty as to how well the aperture averages are computed. Patches of area within the apertures that were near the center were covered approximately five times by the probe beam. Portions near the rim of the aperture were covered no more than once. This center-weighting would tend to bias the average if the responsivity varied radially, which Fig. 13 shows to be the case at 500 nm for Photometers 2 and 3. While the uncertainties due to nonuniform responsivity are difficult to quantify, given the typical magnitudes shown in Figs. 12b and 13, we estimate that the nonuniformity causes an additional 0.02 % relative standard uncertainty in determining *s*(*λ*).

**Fig. 16:**
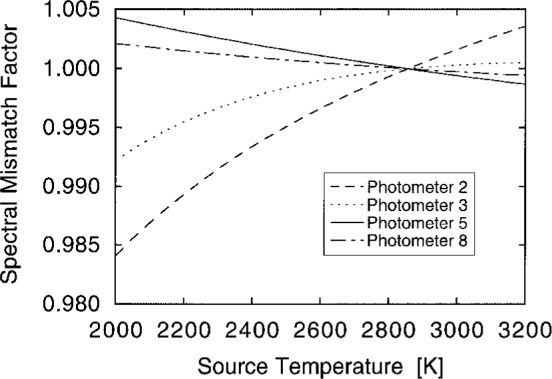
Effect on photometer calibration when sources at different temperatures *T* are viewed. The required correction is reported as *F*(*T*)/*F*(2856 K). Representative packages: Photometer 2, dashed curve; Photometer 3, dotted curve; Photometer 5, solid curve; Photometer 8, dash-dot curve.

When an actual lamp is used, its color temperature may be other than the desired 2856 K or its spectrum may be other than true Planckian. Figure 16 shows the sensitivity of *F* to variations in blackbody temperature for the different types of filters used. For an uncertainty in the temperature of ± 10 K, the uncertainty in *F* amounts to no more than 0.02 %. To quantify the non-Planckian effect, we measured the spectral irradiance of five inside-frosted lamps of the type formerly issued by NIST for luminous intensity standards. While their correlated color temperatures were ≈ 2850 K, their distribution temperatures were within 3 K. Equation (5) was evaluated for each photometer and for each lamp using its actual spectra, and the results were no more than 0.02 % greater than when presuming a 2856 K blackbody.

The evaluation of *F* does not include infrared and ultraviolet response beyond the domain of *V*(*λ*). However, each is a potential problem. Evaluation of Eq. (2) using the spectral responsivity data of Fig. 12a shows that the infrared response (800 nm to 1100 nm) is less than 0.003 % of the signal for a 2856 K radiator. Ultraviolet response (200 nm to 400 nm) is less than 0.002 %.

Two experimental factors characteristic of the SCF affect the responsivity calibration through both *s*(555) and *F*. First, the integral in Eq. (2) is dependent on the wavelength calibration of the SCF. Numerical simulation using the responsivities of the photometers (Fig. 12c) and 2856 K blackbody sources shows that *F*/*s*(555) varies by 0.69 %/nm of offset. The wavelength calibration uncertainty of 0.2 nm leads to an uncertainty of 0.14 % in the calibration of the photometer.

Second, *s*(*λ*) measurements can be affected by the angular convergence (to a focus) of the probe spot. The optical density of the filter would appear too large when a light ray from the monochromator intersects it obliquely, giving an erroneously low value of *s*(*λ*). While the photometer is aligned normal to the beam axis within a few milliradians by retroreflecting the alignment laser shown in Fig. 5, the lamp sources are focused using *f*/9 optics, which have a maximal angle of incidence of 55 mrad. Presuming the sole effect of the filter is absorption, excluding front-surface reflection, the proportionately longer path length at that angle for the data in Fig. 9 would bias the integral in Eq. (2) by 0.20 % (Photometers 1 and 2, the worst case). The actual bias would be less, considering the distribution of angles within the ray bundle and the reflection that was ignored. Since the bias varies as *θ*^2^, a uniform distribution of rays would give an overall bias of 0.10 %.

To mitigate these two effects and to improve accuracy, we used both the SCF and the NIST Reference Spectrophotometer [38] to measure the transmittance of the *V*(*λ*) filters. Comparison of the data, matching peak position and shape, indicated that the two sources of bias on the SCF fortuitously canceled each other. The residual uncertainty in the responsivity caused by the wavelength scale is 0.04 %, and that caused by the SCF optics is 0.05 %.

The Precis 3000 aperture area measurements, for Eq. (7), are given in Table 3, and their uncertainty is included in Table 2. While these measurements were made while the apertures were detached, we also sought to confirm their behavior when they were installed in the photometers. For this, we used the SCF. Consider the output light beam from the monochromator as having a principle axis and an irradiance *B*(*x*′, *y*′) (W/m^2^) in a plane more-or-less perpendicular to this axis, its coordinate origin at the intersection point. The photometers were mounted on the *x*-*y* carriage, in order to position the probe beam axis at point (*x*, *y*) in the aperture plane. If *s*(*x*, *y*) is the responsivity *s*(*λ*) of the photometer at (*x*, *y*) with a wavelength setting *λ* of the monochromator, the total signal from the photometer

$$I\left(x,y\right)=\int \int_{-\infty }^{\infty }s\left(x+x\prime ,y+y\prime \right)B\left(x\prime ,y\prime \right)dx\prime \text{dy}\prime .$$ (9)

Using the *x*-*y* carriage, the probe beam can be scanned over the photometer in fine steps, and the output summed, approximating

$$\begin{array}{c} \int \int_{-\infty }^{\infty }I\left(x,y\right)dx\;dy=\\ ∭\int_{-\infty }^{\infty }s\left(x+x\prime ,y+y\prime \right)B\left(x\prime ,y\prime \right)dx\prime \;dy\prime \;dx\;dy\\ =\int \int_{-\infty }^{\infty }\left[\int \int_{-\infty }^{\infty }s\left(x+x\prime ,y+y\prime \right)dx\;dy\right]B\left(x\prime ,y\prime \right)\;dx\prime \;dy\prime \\ =\int \int_{-\infty }^{\infty }s\left(x,y\right)dx\;dy\int \int_{-\infty }^{\infty }B\left(x\prime ,y\prime \right)dx\prime \;dy\prime . \end{array}$$ (10)

(The separation follows after transforming the inner integral, *x*→*x* – *x*′.) The first integral on the right is the product of the aperture area and the average photometer responsivity within that area. It is the important quantity for any sort of irradiance measurement instrument, including the photometer described in Eq. (7). The second integral is just the total beam power *B*(W). Given an independent determination of the average *s*(*λ*) within the active area of the aperture, by completely overscanning the aperture with small step size Δ*x* and Δ*y*, the aperture area *A* is given by

$$A=\frac{\sum I\left(x,y\right)\Delta x\;\Delta y}{B\;s\left(\lambda \right)}.$$ (11)

This fine scanning was, in fact, the exercise reported in connection with Figs. 12b and 13. Such area computations, averaged over wavelength, are also shown in Table 3. The uncertainty due to the scatter of the data of different wavelengths is shown as well.

It is clear that there is an unresolved discrepancy between the two methods. It cannot be accounted for solely by temperature variations, the residual uncertainty in the average responsivity, or the reliability of the displacement measurements Δ*x* and Δ*y*. Numerical modeling indicates that a small portion of it may arise from reflections and scattering within the photometer, where the back side of the aperture traps light that would otherwise escape. The discrepancy does not cast doubt on the actual aperture areas, as the Precis 3000 measurements differed on average by only 0.01 % from independent measurements made by the aperture manufacturer. Either the problem lies in this second method of determining areas, or there may be an unaccounted aspect of the photometers themselves. An additional uncertainty component of 0.12 % is included in the uncertainty budget to account for this and other possible influences.

**Fig. 17:**
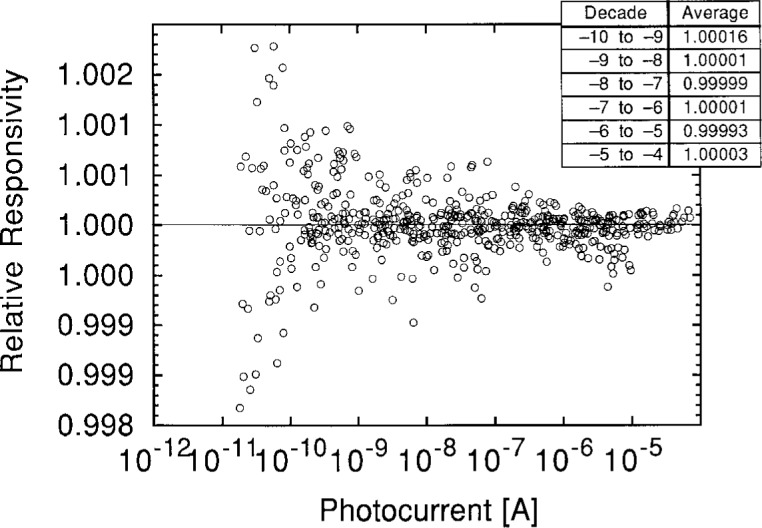
Relative responsivities of Photometer 2 as measured with the beam conjoiner at various input powers.

Additional small uncertainties arise from the method of temperature-correcting the photometers (0.03 %), from potential polarization selectivity of the photometers (0.01 %), and from the electrical calibration of the amplifier (0.003 %). There is also an uncertainty in the calibration due to a potential nonlinear response of the photometers, that is, whether the output voltage remains proportional to the illuminance for disparate values of the same. We presume that the answer is spectrally independent, or at least insensitive to the color temperature of an incandescent lamp that is attenuated by “neutral” density filters. Figure 17 shows the results of a linearity test on a typical photometer using the beam conjoiner method previously described [39]. During calibration, the photocurrent peak (at 555 nm) is typically 10^−6^ to 10^−7^ A. Clearly, nonlinearity effects contribute an error of less than 0.001 %.

## 4. Realization of the Candela

### 4.1 Photometry Bench

**Fig. 18:**
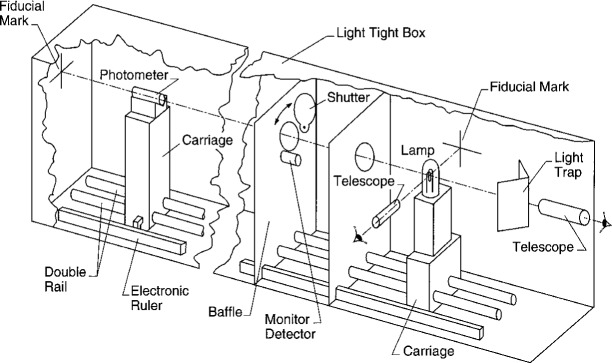
New NIST photometry bench.

The application of a photometer, measuring illuminance, to the luminous intensity determination of a light source [Eq. (8)] is facilitated by the optical bench shown in Fig. 18. The base consists of three 1.8 m (6 ft) long, 46 cm (18 in) thick, steel optical tables with a regular array of tapped holes. Upon it, rigid telescope mounts and upright, marked fiducial plates define the reference axes. The longitudinal axis runs parallel to rails upon which a carriage glides, holding a photometer. A support with cross hairs is substituted for the photometer to align the carriage and rails; lateral alignment within ± 2 mm is achieved at the end opposite the telescope. By substituting a flat mirror for the photometer and by viewing the telescope in itself, orthogonality is ensured to within 5 mrad. A lamp being measured is mounted on another carriage, which permits it to be placed at the intersection of the reference axes. With a side-viewing telescope, the lamp filament is aligned to the plane defined in combination with the vertical fiducial mark. (When frosted lamps are measured, such as the type previously issued by NIST as luminous intensity standards, a model is aligned rather than the lamp itself. The model contains additional fiducial marks both to set the filament plane and to locate the filament within that plane [7].)

The lamp is powered by a constant-current source, which is set under computer control with a resolution of 0.15 mA. The current is independently monitored across an air-cooled, Leeds & Northrup 4360, 0.1 Ω precision shunt resistor [40], which is calibrated at NIST under operating conditions with a standard uncertainty of 0.002 %. The proper operating current for the color temperature of interest is determined by repeated measurements using a diode-array-type spectroradiometer. Additionally, the computer monitors the lamp voltage and the photometer signal and temperature, and it operates the shutter under programmed control.

The apparatus in Fig. 18 is covered by a plastic box lined with black velvet. Surfaces within the box, to the maximum extent possible, are either painted black or covered with black cloth. A baffled chimney above the lamp permits convective cooling without introducing stray light. A light trap is interposed in front of the longitudinal telescope during operation to minimize the light that is reflected back at the photometer. (The side telescope is blocked by black cloth.)

To estimate the magnitude of stray light resulting from reflections and scattering, an additional photometer was used concurrently during testing and evaluation. It was placed outside the area illuminated through the baffles, but near, and oriented in the same general manner as, the photometer being used for measurement. With various arrangements, the stray light was consistently < 0.03 % of the signal. To estimate the stray light originating near the lamp, we covered the side of the lamp towards the photometer. This signal was < 0.001 % of the original. The box attenuated the ambient light from the laboratory by a factor on the order of 10^6^.

### 4.2 Lamp-to-Photometer Distance

The position of the photometer carriage is monitored by a computer-readable, absolute linear encoder with a resolution of 0.013 mm. The distance *r* between the photometer and the transverse reference axis, and a lamp filament, is fixed by sliding an attachment on the photometer carriage into the view of the telescope so that the zero position can be noted. The accuracy of the encoder was checked with a 2.75 m (9 ft) vernier caliper by moving the photometer carriage to various positions and measuring its distance mechanically from the telescope mount as well as electronically. These repeated measurements had a consistency between the methods of 0.18 mm, which we take to be the uncertainty in determining the distance. In actuality most of this scatter was associated with the use of the large caliper, and it will not affect photometric measurements. A standard uncertainty of 0.18 mm in separation corresponds to a relative standard uncertainty in luminous intensity of 0.01 % when the photometer is 3.6 m from the lamp at the far end of the bench.

More significantly, a lamp is not the point source envisioned in Fig. 2c. The size of the radiating volume requires that *I*_v_ in Eq. (8) be taken as the asymptotic value at large *r*. Typical inside-frosted lamps calibrated at NIST are tubular with a radius of 5 cm and extend 10 cm below the center of the filament, which is 5 cm below the top of the lamp. Less important is the transverse extent of the radiating and scattering surfaces, away from the longitudinal axis. At a distance of 2 m to the photometer, a lateral displacement of 10 cm by a point source would decrease its reading by only 0.38 % (0.25 % because of the increased distance and 0.13 % because of the increased angle of incidence). In comparison, a 5 cm longitudinal displacement of a point source would affect the reading by 5 %. Clearly the model is most sensitive to the longitudinal location of the origin of the light.

For this study, the automation afforded by computerized instrumentation and data analysis permitted us to make rapid measurements with the photometer at many distances from the lamp. In this way, an effective origin of the light was found as the best-fit offset *r*_o_ in the expression

$$E_{v}=\frac{I_{v}}{{\left(r-r_{o}\right)}^{2}},$$ (12)

given the measured illuminance *E*_v_ as a function of *r*. (Similarly, the best-fit luminous intensity *I*_v_ can be derived.)

Five inside-frosted lamps were measured in this fashion, each with two randomly chosen photometers. The intensity of the lamp was monitored during these measurements by a stationary, unfiltered, temperature-controlled silicon photodiode. It was exposed to the lamp through a fiber-optic cable, the other end of which was mounted on the second baffle where shown in Fig. 18. The photodiode assembly itself was shadowed from direct radiation from the lamp. This data was used to compensate the output of the moving photometer for variations in the lamp intensity. Equation (12) was best fit by including only data taken with *r* between 270 cm and 370 cm, the maximum of the apparatus.

Typical offsets of 0.50 cm ± 0.15 cm were found for NIST inside-frosted lamps, with a systematic tendency for the offset to decrease by ≈ 0.15 cm after a lamp had been burning for ≈ 1 h. This may be attributed in part to imperfect compensation by the monitor if the spectral distribution of the lamp was changing, particularly in the infrared. Surprisingly, similar offsets of 0.3 cm ± 0.2 cm were found in a set of five, unfrosted Osram WI 41/G lamps. However, part of this (< 0.2 cm) can be attributed to the shape and thickness of the glass envelope, which, acting as a diverging lens, displaces the apparent position of the filament.

The uncertainty of *r* in Eq. (8) is dependent both on the physical measurement of distance and on the applicability of the model Eq. (8) represents, that is, on how one wishes to treat the issue of the effective origin of the light. To ignore it means including a potential systematic error in *r*; to measure it means using up precious hours of a standard lamp’s life. For the purpose of defining the new NIST scale of luminous intensity, we presume that the offset is determined and applied, either for the lamp being measured or from a collection of lamps of similar construction. The relative combined standard uncertainty of *r*, *u*_c,r_(*r*), is then dominated by the uncertainty in the offset distance, typically 0.11 cm in our measurements. At *r* = 3.7 m, the corresponding relative uncertainty in luminous intensity is 0.06 %.

### 4.3 Self-Consistency of Photometer Group

The calibration errors due to random causes can be established for the photometers by measuring the same luminous intensities with all of them, under the same conditions. This was done with a group of five insidefrosted standard lamps, and the results are shown in Table 4. Some photometers gave results consistently above or below the group average for every lamp. This is because what were random effects during calibration become “frozen” into the responsivity assignment for each photometer. However, we can average out this variation by applying correction factors to the original calibrations in order to bring the set of calibrations into self-consistency. Such correction factors are given in the table.

The correction factors are calculated by modeling each entry in Table 4 as the product of a true luminous intensity for the lamp in that column (five unknowns) and a correction factor for the true photometer responsivity in that row (eight unknowns). These 13 values are derived by data fitting; the full procedure will be published separately. In effect, each photometer calibration is compared with the average of them all, and each is slightly adjusted such that the adjusted values do not bias the group average. Strictly, the normalization condition for the correction factors is that their product must be 1. The results show that the random effects that arose during the calibration of the photometer responsivities affected the calibrations, on average, by 0.15 %. The residuals after the data fit show that the random error in making each luminous intensity measurements for the table had a relative standard deviation of 0.02 %.

The scatter in Table 4 can be reduced to 0.11 % by using the aperture areas measured by the SCF found in Table 3 for Eq. (8), but this may be deceiving. Photometers 1 and 2 not only have the larger (hence better known) aperture areas, they also require the most severe uniformity corrections (Fig. 12b); this indicates a potential bias in this alternative.

The same experiment was repeated with a set of five Osram WI 41/G lamps. The correction factors were found to be the same within 0.05 %, except for Photometer 6, which was different by 0.1 %. The residuals had a relative standard deviation of 0.06 %. Since the inside-frosted lamps appeared to be better behaved, we henceforth apply the correction factors in Table 4 to the calibrations in Table 1 for routine use of individual photometers. The additional consistency between the groups of two different types of lamps was most encouraging.

The result that the calibrations of a set of photometers had an actual random standard deviation of 0.15 % may be compared with Table 2. Random influences noted in the Table 2 uncertainty budget (those of Type A, and some fraction of the uncertainties in aperture area and temperature) together amount to a relative standard uncertainty ≈ 0.06 %. The difference is surprising, and is perhaps the result of 1/*f* noise in one of the measurement steps. However, in the end the conclusion of Table 2 is still meaningful. The random component of each photometer after averaging (the self-consistency correction) would have a relative standard deviation of $\left(0.15/\sqrt{8}\right)\%$, which is also ≈ 0.06 %. That is, the combined relative standard uncertainty in Table 2 should be taken as applicable following the self-consistency step just described.

### 4.4 Uncertainty Budget for Luminous Intensity Measurements

In Table 5 the uncertainties for luminous intensity measurements of inside-frosted lamps are summarized. The starting point is the uncertainty budget in Table 2; *u*_c,r_ for the illuminance responsivity of a photometer, 0.19 %, carries over directly and becomes the dominant uncertainty in this budget. The measurement noise contributes 0.02 %, as explained in Sec. 4.3.

The photometers are operated through three cycles of exposure and darkness. Each period of exposure or darkness is ≈ 3 s, including settling time and an integration time of 1.67 s for the output voltage measurement. This provides sufficient noise reduction, yet is sufficiently quick to obviate worry about heating the filter because of optical absorption, a mechanism that would not be detected by the temperature probe. While a precise model would depend on detailed knowledge about the construction of the filters, we can demonstrate an order-of-magnitude estimate. Presuming that all power dissipated from a 500 W lamp is radiated, at a distance > 2 m the irradiance is < 4 mW/cm^2^. Taking a typical specific heat of glass to be ≈ 1700 mJ/(K · cm^3^) and an optical depth of a temperature sensitive, thermally insulated, totally adsorbing layer to be ≈ 0.1 cm, a 3 s exposure would raise the temperature of this layer by ≈ 0.07 K. These severe assumptions show that the influence of absorption on one measurement is < 0.006 %. While any short-term drift of the photometer cannot be attributed to absorption by the filter at these power levels, errors might arise at higher irradiances or with longer integration times. (Possible tracking errors of the thermometer in an environment with a slowly changing ambient temperature were taken into account in the Table 2 uncertainty budget.)

The uncertainties of the photometer to lamp distance, *r* in Eq. (8), are discussed in detail in Sec. 4.2. There is a 0.06 % relative standard uncertainty in luminous intensity measurements resulting from the difference between the geometric and effective position of the lamp filament. The relative standard uncertainty caused by the electronic ruler is < 0.01 %.

The various geometrical uncertainties make negligible contributions to the overall uncertainty. A transverse misalignment of the photometer by ± 2 mm would affect the measurement by only a few parts in 10^7^. A nonorthogonality to the longitudinal axis of 5 mrad would affect the measurement by < 0.002 %. Clearly the geometrical prerequisites of Eq. (8) are met. The angles of incidence on the photometer from the extended source are much less than those encountered during illuminance calibration, and this would tend only to reduce the possible systematic error in numerical aperture already accounted for.

NIST originally elected to use inside-frosted lamps as luminous intensity standards because measurement results were less affected by small changes in the orientation of the lamps [41]. Variations of < 0.2 % were reported for misorientations in pitch (about the vertical lamp axis) of less than ± 2°. Similarly, the fine-grained frosting aids in generating uniform illuminance in the far field, in the neighborhood of the photometer. We believe that any remaining local variations in illuminance will not contribute to possible measurement error beyond those already accounted for in connection with the spatial averaging of the responsivity of the photometers. Errors that may arise because of the differences in lamp orientation between NIST and other laboratories are beyond the scope of this paper.

At the operating point, marginal fractional changes in lamp current cause magnified fractional changes in lamp output by factors of 6 to 8 [42,8]. Since the nominal current of an inside-frosted lamp is 3 A, the 0.15 mA resolution in the current control implies a luminous stability of 0.02 %. The 0.002 % calibration relative standard uncertainty of the shunt resistor implies a reproducibility in output of 0.016 %. Together these imply a relative standard uncertainty component resulting from lamp current measurement of 0.03 %.

Before luminous intensity measurements were made, the lamp currents were ramped slowly up to the operating point, and the lamps were allowed an equilibration time of at least 10 min. Nevertheless, it is important to remember that lamps change with age rather than reach a stable equilibrium. Figure 19a shows the behavior of three types of lamps over the course of 2 h of operation. The scatter in the data, or noise, was discussed in connection with Table 4. Figure 19b demonstrates that the effect spans separate lamp lightings. The gaps in the data correspond to ramping and equilibration periods during which no data were taken. While Fig. 19a shows that the lamps changed most rapidly for an additional 20 min to 30 min after the initial warm-up period (as noted above in connection with the determination of *r*_o_), permanent changes in luminous intensity of 0.1 %/h contraindicate long equilibration times and are a severe limitation on a calibration service requiring lamps as transfer standards. More recently, modified FEL 1000 W quartz-halogen lamps were further tested for suitability as photometric transfer standards, and they were shown to be stable to within 0.2 % − 0.6 % over 60 h of operation [43].

### 4.5 Comparison of New and Old Scales

Before this study the last full realization of the old luminous intensity scale (Fig. 1) occurred in 1985 in connection with the international intercomparison of such scales [44]. At that time the NBS candela was found to be 0.58 % smaller than the world mean. (That is, lamps calibrated at NBS were given higher candela values than the average.) Of this, 0.35 % was later removed with the adoption of ITS-90 [20], making the NIST scale 0.23 % smaller than the world mean.

Encouraging early results by Andor and Zalewski in 1988 [45] showed that a detector-based candela gave results 0.07 % larger than the world mean. This was determined by measuring the primary lamp group with the prototype photometers similar to those reported in this study. Based on this and other indirect evidence, in Ref. [22] we concluded that the new scale realization described in this study did not cause a significant scale shift in comparison with the uncertainty of the old scale, and that it was perhaps on the order of 0.3 %.

While studies continue at NIST to validate this result, additional confirming evidence has recently become available. In 1985, the luminous intensity scale of Germany maintained at the Physikalisch-Technische Bundesanstalt (PTB) was found to be 0.32 % larger than the world mean [44]. A comparison of the new NIST scale with the PTB scale [46] showed that the scale difference narrowed from 0.9 % in 1985 to 0.2 % in 1993. This implies that the new NIST scale is 0.12 % larger than the 1985 world mean, a 0.35 % shift from the old NIST scale with the ITS-90 correction applied. Additionally, the Országos Mérésügyi Hivatal (OMH) in Hungary has maintained a scale based on the BIPM lamp group that holds the 1985 world mean. Preliminary data from a comparison of the new NIST scale with the OMH scale implies that the NIST scale is 0.03 % smaller than the world mean, a 0.2 % shift from the old NIST scale. Another international intercomparison is planned for 1995 [47].

### 4.6 Long-Term Stability of the Standard Photometers

The calibration procedure described in Secs. 3.5 and 4.3 has been repeated twice to test the stability of the calibration result shown in Table 1. The results are shown in Table 6. For the purpose of comparison, the data are adjusted to correspond to a uniform temperature of 298 K and normalized to the calibration values in Table 1. The data shows that the group average changed by < 0.1 % in their first year, and then by an additional 0.4 % in the subsequent 2 years.

One reason for this change appeared to be a surface film that had developed on the exterior side of the glass filters on Photometers 1 and 2. These filters were wiped gently with dry lens tissue, and their photometers were recalibrated. Indeed, their values shifted significantly. The average drift of Photometers 3 to 8 remained ≈ 0.11%/yr.

## 5. Conclusion

Two major goals have been reached. A luminous intensity scale has been derived with detectors, and in a simpler and more direct manner than before. In the process the uncertainty of lamp calibration has been reduced.

This change also puts NIST on good footing for future improvements. The principal uncertainties in the illuminance calibration, the uncertainty of the spectral responsivity scale and the uncertainty in the aperture area, will be reduced significantly by ongoing research and development in our Division. We can expect to reduce the smaller uncertainties as well by improvements in measurement technique. A 0.2 % relative expanded uncertainty (*k* = 2) in illuminance measurement appears to be achievable.

Based on our experience, we believe that the detector-based scale will prove more durable and stable than the lamp-based scale. Nevertheless, yearly recalibration of the standard photometers will be required to maintain the accuracy of the scale, and frosted FEL lamps hold promise as an improved vehicle for disseminating the scale.

This study is of particular benefit for those many applications where illuminance needs to be measured directly, including imaging (such as photography) and ergonomics, where the effects of lighting rather than the light sources themselves matter. The standard photometers have enabled NIST to expand its range of services to include the calibration of luminance meters and illuminance meters [48]. In the field, secondary-standard illuminance meters can be used to calibrate other illuminance meters by substitution, eliminating the need for a long optical bench. Further, the standard photometers have been applied to realize a detector-based geometrically total luminous flux scale for the measurement of lamps [49]. This important development brings the benefits of this study to the lighting industry, for which total luminous flux is perhaps the most important measurable quantity.

While traditional photometry has always involved standard light sources, e.g., lamps in recent decades, detector-based standardization permits smaller uncertainties and often simpler procedures. Unlike lamps the photometers require no large power supplies, and they are useful over a wide dynamic range. Photometry benches need not be long to provide for 1/*r*^2^ attenuations. Well-characterized photometers should prove especially useful for the calibration of modern, non-incandescent light sources, including self-luminous displays. (Care needs to be taken to know the spectrum of the source.) Stable photometers also permit the incidental use of lamps during calibration procedures without regard to their long-term stability. With standards-quality lamps difficult to procure, this alternate technology merits particular attention.

## Figures and Tables

**Fig. 9a:**
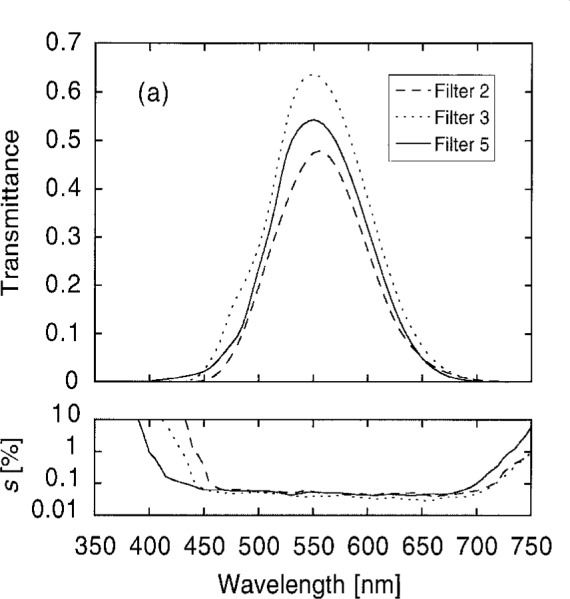
Transmittance of the matching filters used in the detectors. The standard deviation of the measurements, as the percent of the signal, is shown. Representative samples of the filters from the three sources: Filter 2, NRC, dashed curve; Filter 3, NPL, dotted curve; Filter 5, PRC, solid curve.

**Fig. 9b:**
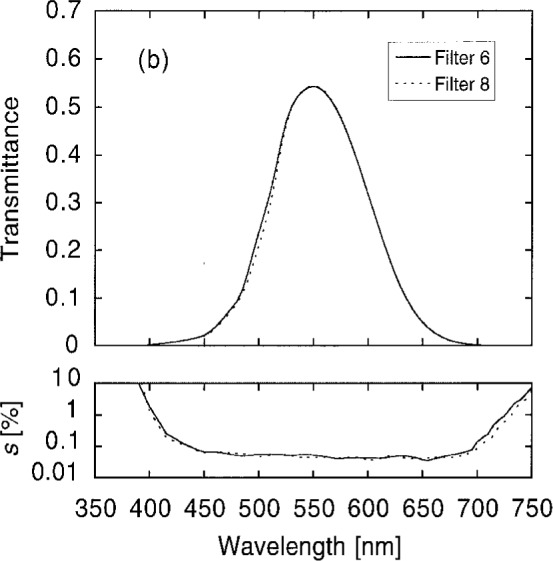
Transmittance of the matching filters used in the detectors. The standard deviation of the measurements, as the percent of the signal, is shown. Comparison of the two batches of PRC filters: Filter 6, first batch, solid curve; Filter 8, second batch, dotted curve.

**Fig. 10a:**
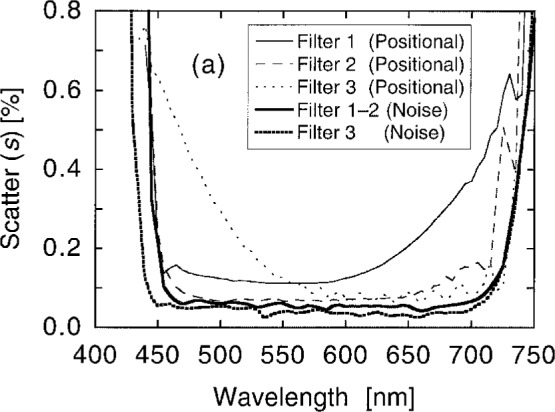
Transmittance uniformity of the matching filters, comparing several positions on the filters. The variation between the measurements is given by their standard deviations from their means. NRC and NPL filters. The heavy curves are the limiting measurement noise: solid for NRC, broken for NPL. Filter 1 (NRC) is the light solid curve; Filter 2 (NRC) is the light dashed curve; Filter 3 (NPL) is the light dotted curve.

**Fig. 10b:**
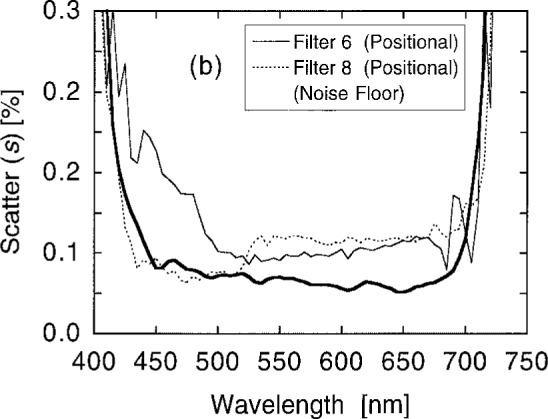
Transmittance uniformity of the matching filters, comparing several positions on the filters. The variation between the measurements is given by their standard deviations from their means. PRC filters. The heavy curve is the measurement noise. Filter 6 (first batch) is the light solid curve, and is typical of the others in the batch. Filter 8 (second batch) is the light dashed curve.

**Fig. 12a:**
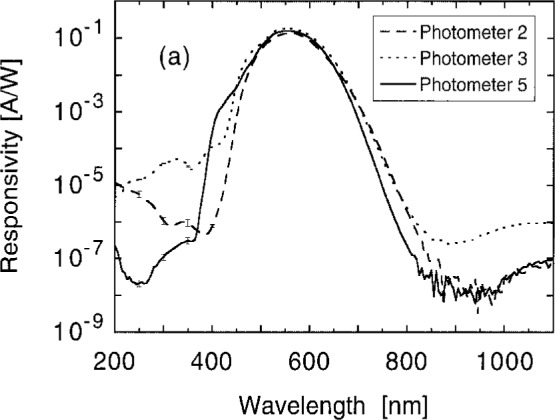
Responsivity of the filtered photodiode packages with emphasis on their behavior in the UV and IR. One spot in the center of the aperture is probed. The measurement uncertainty at this spot is commensurate with the width of the curve in the visible, with the apparent scatter of the data in the IR, and shown by error bars in the UV. Representative packages: Photometer 2, NRC, dashed curve; Photometer 3, NPL, dotted curve; Photometer 5, PRC, solid curve.

**Fig. 12b:**
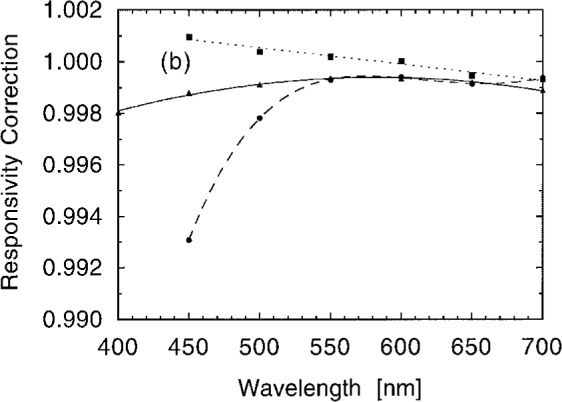
Comparison of responsivity at the center spot with the average of many spots over the face of the aperture. Data taken at 50 nm intervals are interpolated by polynomial fits. The correction factor converts the responsivity at the center to the average responsivity over the face of the aperture. The curves are as in Fig. 12a.

**Fig. 12c:**
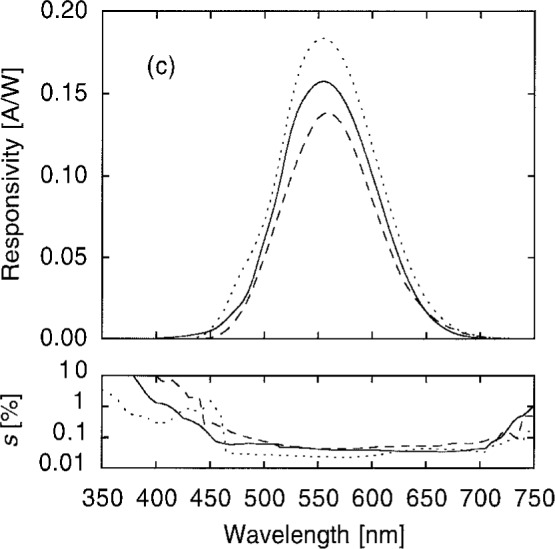
Responsivity of the filtered photodiode packages. The curves are as in Fig. 12a, after the corrections in Fig. 12b have been applied. The standard deviation of the measurements is shown below.

**Fig. 19a:**
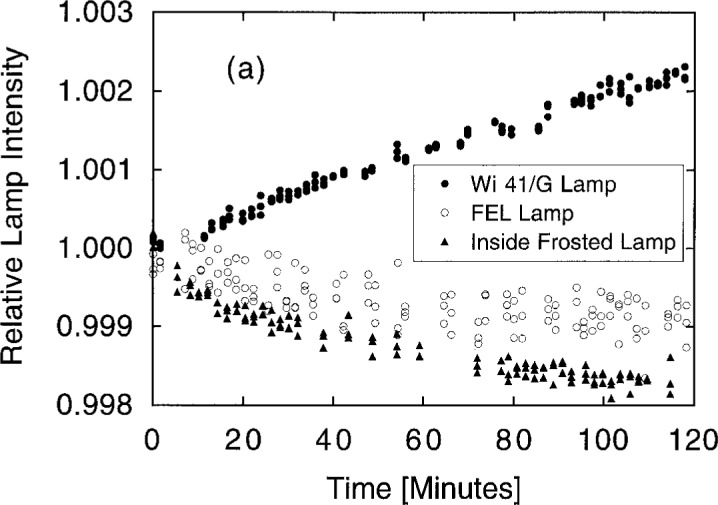
Drift and noise in the output of representative standard lamps during one lighting of an: Osram Wi 41/G lamp, ●; FEL lamp, ○; and Inside-frosted T-20 lamp, ▲.

**Fig. 19b:**
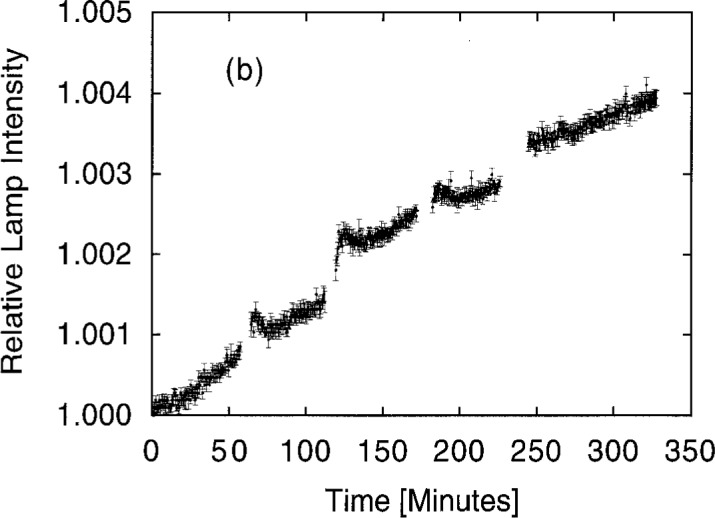
Drift and noise in the output of representative standard lamps during five consecutive lightings of the Osram lamp.

**Table 1:** Summary of the photometers

Photometer	Photodiode	Shunt resistance (GΩ)	Filter source	Calibration (nA/lx)	*F* (2856 K)	*f*_1_′ (%)
1	S1227-1010BQ	5	NRC	10.116	1.002	6.00
2	S1227-1010BQ	5.2	NRC	10.067	1.003	5.97
3	S1226-8BQ	7	NPL	2.821	0.954	7.26
4	S1227-66BQ	6.6	PRC	2.350	0.990	2.55
5	S1226-8BQ	7	PRC	2.335	0.989	2.35
6	S1226-8BQ	7	PRC	2.331	0.990	2.37
7	S1226-8BQ	7	PRC	2.341	0.987	2.79
8	S1226-8BQ	4.3	PRC	2.334	1.000	1.43

**Table 2:** Uncertainty budget for illuminance calibration

Source of uncertainty		Relative standard uncertainty (%)	
		Type A	Type B
*s*(555)			
	Spectral responsivity scale		0.11
	Comparison of photometer with scale	0.04	
*F*			
	Measurement scatter (noise)	0.01	
	Data fitting procedure	0.01	
	Residual non-uniformity within aperture	0.02	
	Color temperature of lamp(± 10 K)		0.02
	Planckian approximation for lamp		0.02
	Infrared leakage		0.003
	Ultraviolet leakage and fluorescence		0.002
Correlated *s*(555) and *F*			
	Wavelength calibration		0.04
	Numerical aperture		0.05
*A*			
	Aperture area (as certified, small apertures)		0.05
Additional			
	Temperature variation		0.03
	Polarization sensitivity		0.01
	Electrical current-to-voltage conversion	0.003	
	Responsivity nonlinearity		0.001
	Other		0.12

Combined standard uncertainty		0.19	
Expanded uncertainty (*k* = 2)		0.39	

**Table 3:** Aperture area measurements

Photometer number	Precis 3000 (cm^2^)	SCF (cm^2^)	Ratio SCF/Precis
1	0.500044 (1 ± 0.02 %)	0.500492 (1 ± 0.03 %)	1.0009
2	0.499756 (1 ± 0.02 %)	0.501015 (1 ± 0.04 %)	1.0025
3	0.099964 (1 ± 0.05 %)	0.100298 (1 ± 0.08 %)	1.0033
4	0.100065 (1 ± 0.05 %)	0.100534 (1 ± 0.05 %)	1.0047
5	0.100042 (1 ± 0.05 %)	0.100375 (1 ± 0.02 %)	1.0033
6	0.099969 (1 ± 0.05 %)	0.100345 (1 ± 0.05 %)	1.0038
7	0.100065 (1 ± 0.05 %)	0.100399 (1 ± 0.06 %)	1.0033
8	0.099857 (1 ± 0.05 %)	0.100206 (1 ± 0.06 %)	1.0035
		Average (3 to 8)	1.0037

**Table 4:** Self-consistency check of photometer group. The luminous intensity (cd) of five lamps are determined with the eight photometers built to realize the scale. Each value was measured three times; the typical scatter was 0.02 % of the mean. The experimental standard deviations of the eight measurements of the lamps, with the different photometers, are given at the bottom. The correction coefficient is explained in the text.

Photometer	Lamp identification number					Correction coefficient
	4975	4976	4977	4978	4979	
1	705.94	707.29	680.34	708.69	708.67	0.9980
2	706.56	707.53	680.92	709.28	709.04	0.9987
3	707.60	709.08	681.70	710.42	710.48	1.0004
4	708.37	709.74	682.66	711.02	711.02	1.0014
5	707.25	708.40	681.27	709.74	709.99	0.9997
6	708.20	709.78	682.85	711.11	710.63	1.0012
7	706.32	707.52	680.39	708.75	708.94	0.9984
8	708.79	710.31	683.22	711.46	711.53	1.0021
*s*	0.15 %	0.17 %	0.17 %	0.15 %	0.15 %	0.15 %

**Table 5:** Uncertainty budget for luminous intensity measurements

Source of uncertainty		Relative standard uncertainty (%)	
		Type A	Type B
Illuminance Responsivity			
	Scale uncertainty from Table 2		0.19
	Measurement noise	0.02	
	Filter absorption		0.006
Lamp to Photometer Distance			
	Size and construction of lamp		0.06
	Physical distance measurement	0.01	
Geometrical			
	Photometer transverse placement		a
	Photometer orthogonality		0.002
Lamp Operation			
	Current regulation		0.03
	Aging (per hour)		0.1

Combined standard uncertainty		0.23	
Expanded uncertainty (*k* = 2)		0.46	

a Too small to list.

**Table 6:** Photometer calibration stability

Photometer	Relative illuminance responsivity			After cleaning
	Nov. 1991	Nov. 1992	Dec. 1994	
1	1.0000	0.9998	0.9939	1.0032
2	1.0000	0.9996	0.9875	1.0056
3	1.0000	0.9960	0.9926	
4	1.0000	0.9991	0.9964	
5	1.0000	0.9988	0.9976	
6	1.0000	0.9999	0.9977	
7	1.0000	1.0010	0.9987	
8	1.0000	0.9997	0.9969	

Average	1.0000	0.9992	0.9952	

## Footnotes

2 As an aid to the reader, the appropriate coherent SI unit in which a quantity should be expressed is indicated in parenthesis when the quantity is first introduced.

3 Current is used as an example; the output might be a voltage instead.

4 PTFE, polytetrafluoroethylene, is more commonly known as Teflon, which is a brand name for such materials.

5 The photometers cannot be compared directly against the cryogenic radiometer since the radiometer requires a laser light source. The parallel surfaces of the optical elements in the photometers might cause errors due to interference effects with such illumination.

6 Certain commercial equipment, instruments, or materials are identified in this paper to foster understanding. Such identification does not imply recommendation or endorsement by the National Institute of Standards and Technology, nor does it imply that the materials or equipment identified are necessarily the best available for the purpose.
